# Supplementary material for: EnzML: multi-label prediction of enzyme classes using InterPro signatures
Source: BMC Bioinformatics. 2012 Apr 25;13:61. doi: 10.1186/1471-2105-13-61 (PMC3483700; doi:10.1186/1471-2105-13-61)
Supplement: Addtional file 5 — The Java code to format the data files, evaluate and predict. The file enzml_java_code.tar.gz contains the Java code used to format database data to ARFF and XML formats, to execute cross and train-test (jackknife) evaluations and to record evaluation results to database. More information is included in the readme.txt file and the Javadoc files. The code can be used with a MySQL database. To use a different database software, other JDBC drivers might be required. [file 1471-2105-13-61-S5.gz › java_code/enzml2011/doc/uk/ac/ed/inf/csb/taweka2010/InstancesFactory.html]

InstancesFactory


---


|  |  |  |
| --- | --- | --- |
| **Package** \| **Class** \| **Use** \| **Tree** \| **Deprecated** \| **Index** \| **Help** | |  |
| **PREV CLASS**   **NEXT CLASS** | **FRAMES**    **NO FRAMES**     **All Classes** |  |
| SUMMARY: NESTED \| FIELD \| CONSTR \| METHOD | DETAIL: FIELD \| CONSTR \| METHOD |  |


---


## uk.ac.ed.inf.csb.taweka2010 Class InstancesFactory

```
java.lang.Object
  uk.ac.ed.inf.csb.taweka2010.InstancesFactory
```

---

``` public class InstancesFactory extends java.lang.Object ```

Generates and fill instances for a weka data set

**Version:**
:   21 Feb 2008

**Author:**
:   Luna De Ferrari luna.deferrari-at-ed.ac.uk

---

**Constructor Summary**

`InstancesFactory(DataSetFactory dataSet)`


| **Method Summary** | |
| --- | --- |
| `java.util.TreeMap<java.lang.String,weka.core.SparseInstance>` | `createInstances(java.util.Set<java.lang.String> instancesNames)`             Generate the data instances |
| `weka.core.SparseInstance` | `fillInstance(java.lang.String thisInstanceName, weka.core.SparseInstance instance)`             fills an instance with all the attribute values |
| `weka.core.Instances` | `fillInstances(weka.core.Instances instances)`             Add the instances filled with data to the data set |
| `DataSetFactory` | `getDataSet()` |
| `weka.core.SparseInstance` | `getInstanceByName(java.lang.String name)` |
| `java.util.TreeMap<java.lang.String,weka.core.SparseInstance>` | `getInstanceNameObjectMap()`             get instances map |

**Methods inherited from class java.lang.Object**

`equals, getClass, hashCode, notify, notifyAll, toString, wait, wait, wait`

**Constructor Detail**

### InstancesFactory

```
public InstancesFactory(DataSetFactory dataSet)
```


**Method Detail**

### createInstances

```
public java.util.TreeMap<java.lang.String,weka.core.SparseInstance> createInstances(java.util.Set<java.lang.String> instancesNames)
```

:   Generate the data instances

    :   **Parameters:**: `instancesNames` - the list of instances names **Returns:**: a map containing the instance name as key and the instance as value

---


### fillInstances

```
public weka.core.Instances fillInstances(weka.core.Instances instances)
```

:   Add the instances filled with data to the data set

    :   **Parameters:**: `instances` - a set of instances (a Weka Instances) **Returns:**: the set of filled instances

---


### fillInstance

```
public weka.core.SparseInstance fillInstance(java.lang.String thisInstanceName,
                                             weka.core.SparseInstance instance)
```

:   fills an instance with all the attribute values

    :   **Parameters:**: `thisInstanceName` - instance name: `instance` - a weka instance **Returns:**: a filled instance

---


### getInstanceNameObjectMap

```
public java.util.TreeMap<java.lang.String,weka.core.SparseInstance> getInstanceNameObjectMap()
```

:   get instances map

---


### getDataSet

```
public DataSetFactory getDataSet()
```

---


### getInstanceByName

```
public weka.core.SparseInstance getInstanceByName(java.lang.String name)
```


---


|  |  |  |
| --- | --- | --- |
| **Package** \| **Class** \| **Use** \| **Tree** \| **Deprecated** \| **Index** \| **Help** | |  |
| **PREV CLASS**   **NEXT CLASS** | **FRAMES**    **NO FRAMES**     **All Classes** |  |
| SUMMARY: NESTED \| FIELD \| CONSTR \| METHOD | DETAIL: FIELD \| CONSTR \| METHOD |  |


---
